# Application of systems dynamics and group model building to identify barriers and facilitators to acute care delivery in a resource limited setting

**DOI:** 10.1186/s12913-020-06014-7

**Published:** 2021-01-06

**Authors:** Fiona Muttalib, Ellis Ballard, Josephine Langton, Sara Malone, Yudy Fonseca, Andreas Hansmann, Kenneth Remy, Peter Hovmand, Allan Doctor

**Affiliations:** 1 Centre for Global Child Health, Hospital for Sick Children, 555 University avenue, Toronto, ON M5G 1X8 Canada; 2 Social System Design Lab, Brown School of Social Work and Public Health, Washington University in St Louis, St-Louis, MO USA; 3 Department of Paediatrics, College of Medicine, Blantyre, Malawi; 4 Brown School of Social Work and Public Health, Washington University in St Louis, St-Louis, MO USA; 5 University of Maryland Medical Center, Baltimore, MD USA; 6 Neonatal and Paediatric ICU, National Pediatric Hospital, Phnom Penh, Cambodia; 7 Departments of Pediatrics and Internal Medicine, Washington University School of Medicine, St. Louis, MO USA; 8 Pediatric Critical Care Medicine and Center for Blood Oxygen Transport and Hemostasis, University of Maryland School of Medicine, Baltimore, USA

**Keywords:** Paediatric emergency care, Paediatric critical care, Group model building, Systems dynamics

## Abstract

**Background:**

Group model building (GMB) is a method to facilitate shared understanding of structures and relationships that determine system behaviors. This project aimed to determine the feasibility of GMB in a resource-limited setting and to use GMB to describe key barriers and facilitators to effective acute care delivery at a tertiary care hospital in Malawi.

**Methods:**

Over 1 week, trained facilitators led three GMB sessions with two groups of healthcare providers to facilitate shared understanding of structures and relationships that determine system behaviors. One group aimed to identify factors that impact patient flow in the paediatric special care ward. The other aimed to identify factors impacting delivery of high-quality care in the paediatric accident and emergency room. Synthesized causal maps of factors influencing patient care were generated, revised, and qualitatively analyzed.

**Results:**

Causal maps identified patient condition as the central modifier of acute care delivery. Severe illness and high volume of patients were identified as creating system strain in several domains: (1) physical space, (2) resource needs and utilization, (3) staff capabilities and (4) quality improvement. Stress in these domains results in worsening patient condition and perpetuating negative reinforcing feedback loops. Balancing factors inherent to the current system included (1) parental engagement, (2) provider resilience, (3) ease of communication and (4) patient death. Perceived strengths of the GMB process were representation of diverse stakeholder viewpoints and complex system synthesis in a visual causal pathway, the process inclusivity, development of shared understanding, new idea generation and momentum building. Challenges identified included time required for completion and potential for participant selection bias.

**Conclusions:**

GMB facilitated creation of a shared mental model, as a first step in optimizing acute care delivery in a paediatric facility in this resource-limited setting.

## Background

Over 6 million children and adolescents worldwide die each year, largely due to preventable and treatable conditions [1]. The vast majority of deaths occur in resource-limited settings (RLS) in low- and middle-income countries where delayed illness recognition and care-seeking are common. Severe illness at presentation is exacerbated by barriers to delivery of optimal acute care, together increasing early hospital mortality [2–4].

Barriers to optimal acute care delivery in RLS have been identified. Structural challenges are common, including poor availability of essential equipment, medicines and diagnostic tests and unreliable electricity and clean water access [5]. Human resources are lacking due to insufficient numbers of healthcare workers relative to patient volume, limited paediatric provider training and high staff turnover [6, 7]. A complex interplay of these factors and barriers exogenous to the healthcare system limit ability to easily identify the issues meriting attention that, if addressed, would lead to systems improvement.

At Queen Elizabeth Central Hospital (QECH), a tertiary care hospital in southern Malawi, previous improvements to acute care delivery have been associated with decreased hospital mortality, such as implementation of Emergency Triage Assessment and Treatment and redesigning patient flow in the paediatric accident and emergency (pA&E) department [8]. Barriers to acute care delivery at QECH persist owing to a high burden of severely ill children relative to hospital resources and personnel. As one of only four public, tertiary care centers in Malawi, subspecialty medical expertise at QECH is more readily available than at the district hospital level, however, these services are in extremely high demand. Social determinants of health in Malawi result in severe illness among children, often with delayed presentation to hospital and, as a result, the demands of patient volume and complexity can exceed the available financial, human, and material resources. This includes limited basic laboratory services and microbiology, bed capacity and acute care capabilities, and equipment and drug availability. Recently, the opportunity arose to re-design both the Paediatric Special Care Ward High Dependency Unit (PSCW HDU) and paediatric accident and emergency (pA&E) to address ongoing challenges.

We used a participatory form of systems dynamics modeling called group model building (GMB) to facilitate shared understanding of underlying system structures and relationships inherent to acute care delivery at QECH. GMB is an approach for involving stakeholders in the process of understanding systems through the endogenous, or feedback lens of system dynamics [9, 10]. GMB involves formal workshops consisting of structured activities or “scripts” that are sequenced to elicit variables and generate progressively more refined qualitative diagrams of the structure of interconnections and feedback loops of system structure [11–16]. Workshop design builds on standard sequences and is adapted as part of the design process of a core modeling team which brings together modelers, substantive experts, and community voices to inform the design of group model building work [9, 17]. The resulting qualitative models use diagramming conventions of causal loop diagrams as well as stock and flow diagrams to represent sources of feedback underlying dynamic system behavior [17].

**Fig. 1:**
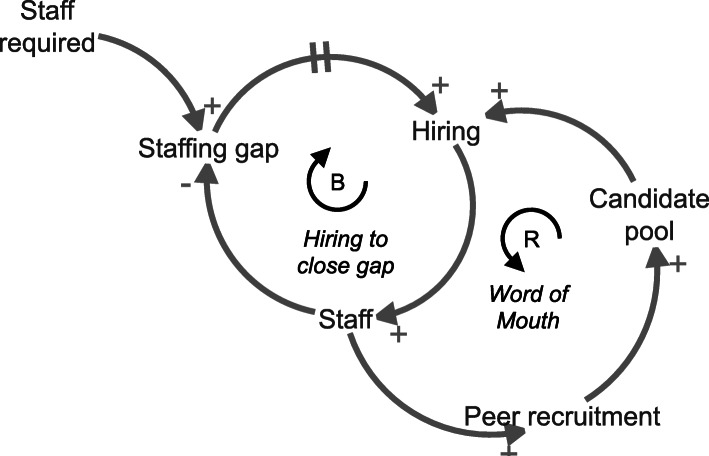
Causal loop diagram describing the interplay between reinforcing and balancing mechanisms. R: Reinforcing, B: Balancing

A central tenet of system dynamics is that system behavior emerges via reinforcing and balancing feedback loops that promote equilibrium or disequilibrium in a system, depending on loop interactions and the strengths of loop inputs. A reinforcing loop denotes a mechanism that amplifies change to the system while a balancing loop counteracts change (Fig. 1) [18]. Examples of reinforcing loops include interest accumulating in a savings account and the adoption of products through word-of-mouth communication. Examples of balancing loops include hiring to close a staffing gap and lowering expectations to meet a goal. Arrows describe positive or inverse causal relationships and double hashmarks indicate delays between input and effect.

While there are some examples GMB being used in resource-limited settings [19–21], few examples exist of its application to clinical interventions. GMB, in contrast with other traditional quality improvement tools, focuses explicitly on the system performance from a dynamic perspective, examining how performance has changed over time as a function of information cues, decision rules, and resource dynamics of the system. This system dynamics perspective explicitly explores how both tangible variables (e.g. patients, equipment, and beds) as well as intangible variables (e.g. stress, trust, and information) are interconnected through feedback relationships, and the role of time delays in influencing their management. This lens of a dynamics feedback system affords opportunities to explicitly examine these factors as potential intervention points or pathways for action that may be ignored or left unaddressed by other methods. We aimed to determine the feasibility of GMB in a resource-limited setting and to use GMB to bring key stakeholders together to identify the barriers to effective acute health care delivery and the areas likely to respond favourably to parsimonious resource allocation.

## Methods

In May 2018, we conducted an exploratory GMB workshop in Blantyre, Malawi at QECH [22]. At QECH, children with acute illness present to pA&E for initial triage, evaluation and resuscitation, if needed. Children may be admitted to one of ten paediatric wards, of which four include an HDU with enhanced monitoring. In the PSCW HDU, available resources include an increased nurse to patient ratio, bubble continuous positive airway pressure ventilation, intravenous infusions, blood transfusion and six hourly vital signs monitoring.

Two facilitators (EB, SM) trained in GMB at the Social System Design Lab at the Brown School (Washington University in St-Louis) led the GMB sessions. Facilitators were introduced to the hospital environment and staff and toured the PSCW HDU and pA&E to understand the basic processes of care. Facilitators held two separate GMB sequences comprising three workshop sessions over 1 week. Two different groups of eight participants attended the PSCW HDU session and the pA&E session. A local paediatrician selected participants for the PSCW HDU sessions which included two registered nurses with both clinical and supervisory roles in the PSCW HDU, one postgraduate trainee, two local paediatricians with expertise in emergency medicine and intensive care, two visiting intensivists and one observer. A local paediatrician and the nurse in charge selected participants for the pA&E sessions and included a paediatric charge nurse, one registered nurse with clinical and supervisory roles, one nurse volunteer, two postgraduate trainees, one clinical officer, one local paediatrician with expertise in emergency medicine and one observer.

In the PSCW HDU sequence, facilitators reviewed basic GMB principles and proposed a seed question for the focus of a variable elicitation “script”: “What factors affect patient flow, or admission and discharge of patients, in the PSCW?” Participants generated a shared list of these factors. Participants then nominated causal links and variables to be added to a digitally projected stock and flow seed structure. The visual representation of the causal structure allowed participants to describe the pathway through which factors influenced rates of inflow and outflow to the HDU and allowed facilitators to probe and elaborate on structural contributions.

The pA&E sequence focused on the question “What are the factors that help or hurt your ability to provide high quality patient care?” In this sequence, participants reviewed GMB principles and generated variables based on the above prompt. Participants worked in groups of 2–3 to build connection circle diagrams that mapped the interconnections between these factors. Using the connection circles as a reference, participants proposed causal links and feedback loops which the facilitation team presented and refined in real time using digital projection.

Following preliminary causal mapping sessions, the facilitators synthesized the raw causal loop diagrams through an iterative process. In the next session, preliminary synthesis causal maps were unfolded for participants for critique and revised in real-time. In the final session, the revised synthesis was used to facilitate a discussion about targets and strategies for potential improvements to PSCW HDU and pA&E. Facilitators collected discussion notes and session artifacts to inform further analysis and interpretation. This project was reviewed by the University of Maryland institutional review board and deemed exempt from review.

## Results

### Paediatric special care ward high dependency unit (PSCW HDU)

**Fig. 2:**
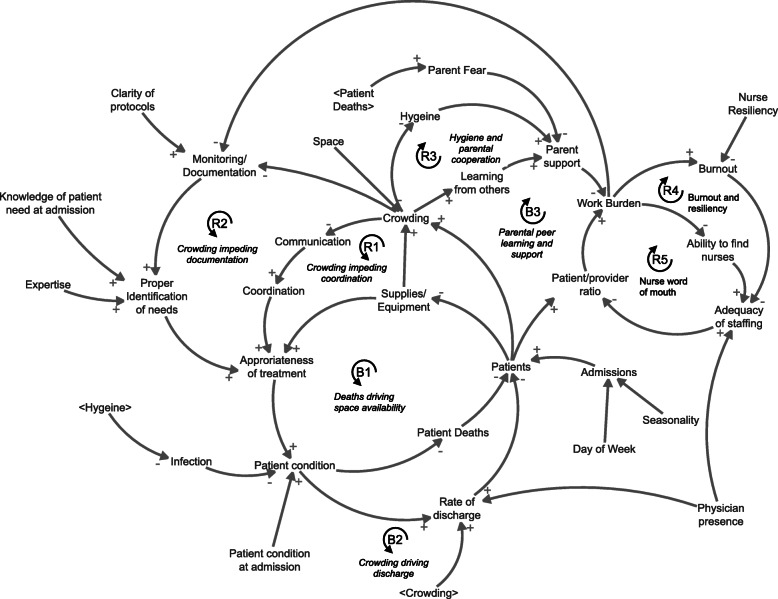
Synthesis of Causal Map of Barriers and Facilitators to Acute Care Delivery in the PSCW HDU. R: Reinforcing, B: Balancing

Figure 2 presents the final synthesis causal map of the underlying feedback structure for clinical care delivery in PSCW HDU. The causal map presents a systems dynamics-based representation of how factors interact to either balance or amplify the effects of patient admissions and increased acuity. Severe illness and high volume of patients stress the system in three interconnected domains: (1) physical space, (2) resource utilization and needs and (3) provider capacity. Strain in these domains negatively impacts care delivery through the activation and perpetuation of reinforcing feedback loops, worsening patient condition and accelerating strain on patient condition. Table 1 summarizes the key feedback loops in the causal loop diagram and Table 2 provides an overview of specific factors identified as potential points of intervention.

**Table 1:** Feedback loops in the HDU model

Loop	Description
R1: Crowding impedes coordination	With limited space, an increase in patients causes an increase in crowding, which impedes communication and coordination, reducing the appropriateness of treatment. Patient conditions are slower to improve, slowing the rate of discharge and thus maintaining crowding.
R2: Crowding impedes documentation	Crowding also reduces the space available for documentation and storage of patient records. This reduction in monitoring and documentation reduces providers’ ability to properly identify and communicate patient needs, thus reducing appropriateness of treatment and slowing patient discharge.
B1: Deaths drive space availability	With rapidly deteriorating patient conditions comes a higher rate of patient mortality. This mortality reduces HDU crowding, forcing an uncontrolled and undesirable balancing mechanism to regain equilibrium in the HDU.
B2: Crowding drives discharge	An increase in crowding forces decisions about how to maintain the operation of the HDU, meaning the risk of discharging patients in order to reduce crowding and maintain functioning capacity of the HDU.
R3: Hygiene and parental cooperation	Increases in crowding reduce the efficacy of hygiene control procedures. Lower hygiene means that parents are less willing to support nurses and physicians in care delivery, increasing the work burden on clinicians and accelerating the adverse effects of crowding.
B3: Parental peer learning and support	Counteracting the adverse impact of crowding on hygiene and coordination, crowding creates opportunities for peer learning and peer support among parents, reducing work burden on clinicians as parents support the care process.
R4: Worker burden	A higher patient/provider ratio increases work burden for clinicians, which increases risk of burnout that further increases the patient/provider ratio.
R5: Nurse word of mouth	A higher patient/provider ratio increases the work burden on clinicians, which makes it harder to find nurses to improve adequacy of staffing. This forms a reinforcing loop where difficult working conditions perpetuate that worker burden through barriers to hiring.

*R* Reinforcing, *B* Balancing, *HDU* High-dependency unit

**Table 2:** Potential points for intervention/redesign in the paediatric special care ward

Key Domain	Factor	Potential or current intervention strategies
Physical Space	Crowding (a function of patients, supplies and equipment, and available space)	Increased bed capacity Formal organizational structures Specialized spaces for documentation, resuscitation, monitoring
	Hygiene	Dedicated isolation areas Implementation of infection control protocols Ensure functioning of handwash stations for visitors and families
	Coordination	Clustering sickest patients together to facilitate communication and treatment
Resource utilization	Supply/equipment	Increase availability of key supplies and equipment
	Tracking and management	Systems for verification of equipment functionality and maintenance System for tracking usage, alerting low supply Systems for checking emergency supply
Staff Capacity	Provider resources	Dedication of senior clinical expertise Scheduling of staff capacity overnight/on weekends Managing competing demands Junior trainee capabilities/training
	Parent support	Parental engagement in patient care
	Provider Resiliency	Strengthening collaborative work ethic Provider retention strategies: Monetary incentives, burnout prevention, hiring
	Coordination	Promote a collaborative work ethic

Two of the feedback mechanisms define ways that crowding impedes coordination, monitoring and documentation of patients (Fig. 2, R1 and R2). Participants identified the main physical space factors driving quality of care delivery to be insufficient bed availability resulting in overcrowding, the absence of a system for allocating, tracking and identifying ward beds at the time of admission, as well as inadequate isolation and hygiene practices. The absence of sufficient designated space for chart documentation and storage was also noted to impede routine care delivery. Last, the advantages and disadvantages of a designated resuscitation area were discussed with respect to optimizing identification and management of acutely deteriorating children, while maintaining the flexibility of the available space. The openness of the space facilitated easy communication between care providers and parent engagement in care delivery.

Resource-related factors were strongly implicated in the reinforcing mechanisms of crowding and hygiene control (Fig. 2, R1, R2, R3). As patient acuity and complexity increases, resources necessary to provide appropriate care likewise increase and available resources are depleted. This depletion potentially clears space and reduces crowding, but lack of resource availability also exacerbates the health status of admitted and incoming patients. Participants noted that an insufficient system for tracking usage, ensuring regular maintenance and maintaining supplies, contributed to low resource availability. In the setting of an emergency, it was also noted that the absence of a designated resuscitation cart and routine maintenance of its contents impaired provider ability to respond rapidly to acute situations.

The final domain of provider capacity describes the relationship between patient volume and problem complexity, and staffing resources and staff capability (Fig. 2, R4, R5, and B3). Acutely ill children require higher intensity of nursing and physician care which may reduce provider availability to care for other patients. In the presence of inadequate staffing, this can contribute to a worsening in the condition of other patients. Inadequate staffing relative to patient needs was thought likely to increase mortality risk on the ward. This, in turn, contributes to staff burnout and decreased staff retention which further exacerbate patient care. High staff resilience in the face of negative outcomes and strong parent engagement were seen as balancing mechanisms. Parental and family support moderated the workload and reduced provider burnout. In conditions of crowding, parents served as resources in treatment, advocating for other parents and children, and helping to orient newly arriving families to the routines and expectations of the HDU (Fig. 2, B3).

### Paediatric accident and emergency (pA&E)

**Fig. 3:**
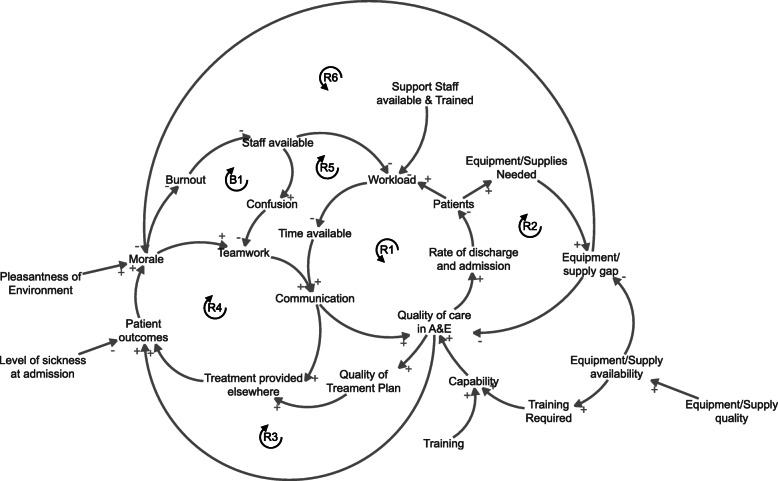
Synthesis of Causal Map of Barriers and Facilitators to Acute Care Delivery in the pA&E. R: Reinforcing, B: Balancing. Physician presence: refers to the physical presence of a supervising consultant physician in the pA&E. Faced with competing academic, administrative, and other demands, a consultant may not always be available and present in the pA&E

Within pA&E, patient volume and condition were core modifiers of acute care delivery (Fig. 3). Participants identified reinforcing factors within this environment in four domains: (1) physical space, (2) resource utilization, (3) staffing, (4) lack of formal quality improvement measures. One balancing factor was identified: provider resilience. Additional exogenous factors not within the control of those in the system were also identified. Table 3 summarizes the key feedback loops in the causal loop diagram and Table 4 provides an overview of specific factors identified as potential points of intervention.

**Table 3:** Feedback loops in the paediatric accident and emergency room model

Loop	Description
**R1: Crowding impedes communication**	Limited space and high patient numbers increases the workload on staff and reduces the ability to communicate which reduces the appropriateness of treatment.
**R2: Patient outcomes drives burnout**	Providing high quality care increases positive patient outcomes, which boosts staff morale and reduces burnout and attrition.
**B1: Staff numbers driving confusion**	As there are more staff available, there is more confusion that occurs between staff which limits communication.
**R3: High morale and teamwork driving quality care**	High morale leads to increased teamwork and communication within the team, which leads to high quality care and better patient outcomes.
**R4: Treatment elsewhere in the facility drive team morale**	Due to the nature of transfer of patients to other units, high quality treatment plans that extend to other units produce positive patient outcomes that boost the morale and further communication of pA&E staff.
**R5: Patient numbers increase supply limitation**	More patients demand greater use of equipment and supplies which create a gap in needed versus available supplies which reduces the quality of care able to be provided.
**R6: Staff burden**	A lack of needed resources reduces morale and places a burden on staff that leads to burnout over time.

*R* Reinforcing, *pA&E* Paediatric accident and emergency

**Table 4:** Potential points for intervention/redesign in the paediatric accident and emergency room

Key Domain	Factor	Potential or current intervention strategies
**Physical Space**	Crowding	Create a dedicated space for education Improve signage to orient patients to pA&E
	Efficient use of space	Addition of designated spaces for radiology, pharmacy Develop flexible spaces for bereavement, adolescent care, short stay patients
	Hygiene	Improve access to hand hygiene supplies (running water and soap or antiseptic)
**Resource utilization**	Equipment	Establish standard process for equipment maintenance and identification Permanently place equipment to ensure security
**Staff Capacity**	Establish roles	Clarify the roles and responsibilities for department staff
	Provider knowledge	Develop opportunities for continued professional development Implement infection control training
	Provider retention	Develop training and onboarding process for new hires Implement strategies to promote provider retention
**Quality improvement**	Communication	Develop communication tools for staff and patients Encourage use of existing suggestion box Implement name badges for all personnel
	Patient flow	Design and disseminate protocols for flow within and out of the department Clear identification of severely ill patients in the department and prior to transfer
	Quality improvement	Design a system for quality audits and improvement

Participants identified space needs specific to the pA&E that significantly impact patient care, including: insufficient number of resuscitation beds, lack of space for counselling, end-of life care and bereavement, absence of an adolescent assessment area, inadequate isolation facilities, non-functioning short-stay ward and non-child friendly environment. These resource needs were viewed as fundamental to being able to appropriately care for patients. They also recognised the need to rethink the use, organization and flexibility of the current space available.

Similar to the PSCW HDU, increased volume of severely ill children in pA&E increases resource utilization in the context of limited resource availability and, in turn, impedes high-quality care delivery (Fig. 3, R2). Participants identified that absence of a system to manage track and maintain resources limited their ability to appropriately manage this resource supply gap. They proposed re-establishing daily checklists for the resuscitation trolley and emergency drugs, consolidating the storerooms and developing a stock list, permanently placing resources to ensure their security and labelling equipment. The inability to track and maintain resources was acknowledged to have a proximal impact on patient outcomes, such as poor hygiene practices posing risk to patient morbidity and mortality.

Increasing patient volume and complexity reduces time available for care delivery. Coupled to staff inexperience or lack of sufficient staff support this amplifies the impact of increased workloads and may worsen staff morale (Fig. 3, R4). Low staff morale increases staff burnout and reduces staff retention, thereby feeding forward in a reinforcing loop (Fig. 3, R5), perpetuating the imbalance between staffing availability and patient volume/complexity. Similar to the PSCW, high provider resilience in the face of negative outcomes was identified as a balancing mechanism. Interventions proposed to balance this sequence included increasing staff availability through review of departmental human resource processes, strengthening staff capabilities and professional development through investment in staff training, encouragement of staff and patient feedback. Similar to the PSCW, high staff resilience in the face of negative outcomes was identified as a balancing mechanism (loop B1).

The imbalance between a high burden of critically ill patients and limited staff availability also negatively impacts communication quality. Sub-optimal communication was noted to negatively affect multi-disciplinary teamwork and patient care; again, composing a reinforcing loop feeding forward to constrain favorable outcomes (loop R1). Participants noted that poor communication between pA&E and external departments affected patient care quality at the time of patient transfer. A need to develop a departmental communication strategy was identified - including name badges, staff board, access to specialty team on-call rosters with telephone numbers and development of communication tools for staff and patients including clear departmental signposting.

Finally, a need for quality improvement initiatives was identified. Inexperience or inadequate training negatively impacts quality of care delivered, directly affecting patient outcome (loop R1). A plan was proposed to recreate a dedicated space for education and establish of a departmental teaching plan. Recommendations were made for introduction of a patient feedback system and use of quality audits to direct improvements in care. A formal scheduled review of patient care was considered important in ascertaining quality.

## Discussion

We used group model building (GMB) to achieve shared understanding of perceived facilitators and barriers to optimal acute care delivery in a resource-limited setting. Though the initial questions centered on how to redesign the PSCW HDU and pA&E to optimize care delivery, the key insight attained was that there were many process of care elements that were as important, if not more important, than physical space limitations. In PSCW HDU, the process highlighted strengths of the existing environment, particularly intangible factors such as collaboration among parents and ease of communication between care providers, as facilitators of performance in the HDU. The role of these factors as facilitators and buffers to system performance are illustrated through the causal loop diagram as one traces how the system may respond to unexpected surges in demand. Crowding, for instance, may have cascading consequences in undermining communications and parental support, begetting rushed or less appropriate patient treatment, compounding challenges of supply and crowding. Understanding the role of these intangible factors in the context of the dynamic feedback system amplified the importance of process of care elements in HDU re-design. In pA&E, GMB highlighted the opportunity to redesign care with existing resources. The workshops and causal map highlight the importance of intangible factors such as morale as a critical component in the cycle of quality care provision in the pA&E, both as a driver of quality care and as an outcome of positive patient outcomes. The causal map suggests the importance of thinking about the reinforcing nature of morale, and how the redesign of physical space might include explicit consideration of these feedback effects, as well as capitalize on existing strengths.

This work highlights the potential of GMB to be utilized in the future to design intervention and evaluation. By developing shared understanding of the tangible and intangible factors that are interconnected to influence system performance, groups can access a wider range of potential leverage points as a focus for intervention. The focus on causal connections and feedback loops can illuminate potential dependencies of resources, and sources of delay gaps in or communication that may be difficult to identify from one stakeholder perspective. This negotiation and visualization opens up the body of potential solutions and use the causal map to trace the multiple consequences in investment in one intervention over another. Further, GMB could be utilized in the future to identify factors within causal chains of system performance and develop process metrics for rigorous evaluation studies.

The GMB exercise proved feasible in this resource-limited context and provided new insights to participants both already working within the system and those with an outsider perspective. GMB offered the following strengths: equal representation of all participant voices, creation of a shared understanding, generation of new ideas, the building of a momentum for change and the ability to identify areas of existing shared mental models and areas of discrepancy. For participants with less familiarity with the system, GMB provided insight as to the complexity of the system and the variety of provider perspectives.

Certain limitations to the use of GMB in this setting were also noted. First, the complete model-building process is relatively time-consuming in a setting where clinical demands are extremely high. Second, there was a risk of selection bias given the methods used to select participants. However, for the purposes of these workshops it was felt these participants were representative of the key stakeholders in both PSCW HDU and pA&E. External validity was enhanced in pA&E through presentation of the causal map to the entire department and further iterative revision to the structure. Third, we acknowledge the importance of staff hierarchy as potentially affecting participant’s willingness to share divergent opinions in the presence of superiors. To circumvent this, GMB approaches often include an additional first step whereby complex teams assemble into peer groups and separately compose causal maps which are then merged to generate a global schematic for unified group review/modification. Fourth, concerns were raised about the cultural translatability of GMB and systems dynamics language. In the accelerated timeline of these workshops, some participants reported difficulty understanding new vocabulary and explanations of the scripted activities. Language, accessibility and cultural adaptation is central to the practice of GMB in community settings where, over time, a collaborative adaptation of the approach is used to improve context relevance. Lastly, the GMB goal was to capture system complexity and, intentionally, there is no implementation component, reducing likelihood of biases related to goals other than generation of accurate system maps. Certain participants questioned the utility of the conceptual schematic in the absence of specific action items to execute. Identification of action items is facilitated by observational validation of the map, empiric weighting of input factors, and formal simulation. While our findings are in keeping with published literature describing barriers to acute care delivery elsewhere [3, 7, 23], our results should be considered specific to the applied setting. It is possible that the structure of the causal loop diagrams would be generalizable to other acute care settings where resources are more consistently available, particularly in a situation of rapid increase in demand (e.g. mass casualty event, the COVID-19 pandemic). The anticipated key differences would likely be the starting values (resources available at baseline, ongoing supply) and the length of delays in the system. Further study of the causal loop diagrams in other settings would be needed for external validation. The GMB process, itself, is adaptive and generalizable to other resource limited settings and confers team benefits other than generation of the actual systems map, itself.

In order to generate actionable goals using the causal map, it was identified that for PSCW HDU, further data regarding patient flow, patient characteristics and resource utilization metrics would be needed. A detailed audit of current practice in the PSCW HDU was undertaken for this purpose. In the pA&E, an audit had recently been completed; therefore, pA&E participants were able to build on the momentum generated from this workshop and implement some of the interventions identified. Specifically, they consolidated storerooms, created a multifunctional isolation, adolescent, and bereavement room, re-instituted checklists for supplies, the resuscitation trolley and emergency drug checklists, recreated a dedicated education area and produced departmental signposts. Lastly, the ideas generated were incorporated into pA&E redesign and subsequent architectural drawings.

## Conclusions

Group model building facilitated creation of a shared mental model in an approach to optimizing acute care delivery in a paediatric facility in a resource limited setting. Strengths of this approach included representation of key stakeholders and synthesis of a complex system into an internally valid causal map. Areas for improvement included the time commitment required for participation in the complete workshop, ensuring representativeness of the participant group, external validation of the causal map with a broad audience and limiting the use of esoteric language. Lastly, clear action items should be generated and followed through to ensure the utility of the group model building exercise.

## Data Availability

Artifacts from the sessions (facilitator notes and preliminary versions of qualitative diagrams) are available upon request to the corresponding author.

## Abbreviations

GMB: Group model building
QECH: Queen Elizabeth Central Hospital
PSCW: Paediatric special care ward
pA&E: Paediatric accident and emergency room
RLS: Resource-limited settings
HDU: High dependency unit
